# Phase II study of weekly paclitaxel and trastuzumab in anthracycline- and taxane-pretreated patients with HER2-overexpressing metastatic breast cancer

**DOI:** 10.1038/sj.bjc.6601485

**Published:** 2004-01-06

**Authors:** S Gori, M Colozza, A M Mosconi, E Franceschi, C Basurto, R Cherubini, A Sidoni, A Rulli, C Bisacci, V De Angelis, L Crinò, M Tonato

**Affiliations:** 1 Medical Oncology Division, Policlinico Hospital, via Brunamonti 51, Perugia 06122, Italy; 2 Medical Oncology Division, Bellaria Hospital, Via Altura 3, Bologna 40139, Italy; 3 Institute of Pathological Anatomy and Histology, Division of Cancer Research-Perugia University, Policlinico Monteluce, via Brunamonti 51, Perugia 06122, Italy; 4 Breast Unit, Surgical Department, Perugia University, Policlinico Monteluce, via Brunamonti 51, Perugia 06122, Italy; 5 Medical Oncology Service, ASL 2 Perugino, Via Piccolotti 1, Marsciano 06055, Italy

**Keywords:** trastuzumab, paclitaxel, metastatic breast cancer

## Abstract

Synergism between anti-HER2 monoclonal antibody (trastuzumab) and paclitaxel has been shown *in vitro* and *in vivo*. In previous experiences, weekly administration of trastuzumab and paclitaxel has shown significant activity in metastatic breast cancer. In this phase II study, we evaluated the activity and the toxicity of this weekly regimen in anthracycline- and taxane-pretreated patients with HER2-overexpressing metastatic breast cancer. Between November 1999 and July 2001, 25 patients were treated with trastuzumab (4 mg kg^−1^ i.v. loading dose followed by 2 mg kg^−1^ i.v. week^−1^) and paclitaxel (60–90 mg m^−2^ h^−1^ i.v. infusion week^−1^). The treatment was planned to continue until disease progression or prohibitive toxicity; in patients with responsive or stable disease, after 6 months of therapy, the decision to stop paclitaxel while continuing weekly trastuzumab was left to the physicians' judgement. At the median follow-up of 19.6 months (range 9.2–38.1), all patients are evaluable for response and toxicity. We obtained four (16%) complete responses (CR), 10 (40%) partial responses (PR), four (16%) stable diseases and seven (28%) disease progressions. The response rate (CR+PR) was 56% (95% CI, 36.5–75.5%). The median duration of response was 10.4 months (range 4.1–24.2+). Median time to progression was 8.6 months (range 2.5–24.2+). The toxicity was mild; five patients experienced fever and chills during the first infusion of trastuzumab (20%); leukopenia grade 2 was recorded in one patient (4%). Two patients (8%) came off study for grade 3 cardiotoxicity (after 9 and 17 weeks of treatment, respectively): both had already received anthracyclines and taxanes. Onycholysis grade 2 was observed in five patients (20%). These results confirm that weekly administration of trastuzumab and paclitaxel is active in anthracycline- and taxane-pretreated metastatic breast cancer patients HER2-overexpressing. Since cardiac disfunctions grade 3 were observed (8%), we recommend that cardiac function should be monitored in these patients.

In the past decade, molecular and genetic changes that cause malignant transformation were identified and these abnormalities can be targets for anticancer treatment. The human epidermal growth factor receptor-tyrosine kinase 2 (HER2) gene is amplified in 25–30% of breast cancers and this amplification causes overexpression of the encoded protein in 95% of cases (Slamon *et al*, 1987). This alteration is associated with more rapid growth of tumour cells and patients with breast cancer overexpressing HER2 have a worse prognosis (Slamon *et al*, 1987; Ravdin *et al*, 1995). Trastuzumab is a humanised monoclonal antibody with specificity for the HER2 protein; it binds to the extracellular domain of HER2 and blocks its function in signal transduction. In preclinical studies trastuzumab inhibited the growth of the HER2-overexpressing tumour cells. Results of phase II trials provided evidence that it is safe and clinically active in patients with HER2 overexpressing metastatic breast cancer, with a response rate of 12–40% (Baselga *et al*, 1996; Cobleigh *et al*, 1999; Vogel *et al*, 2002).

Preclinical studies have shown a potentially enhanced antitumour activity when trastuzuamb is combined with traditional chemotherapeutic agents. In Slamon's experience, the addition of trastuzumab to chemotherapy (doxorubicin plus cyclophosphamide or three-weekly paclitaxel) was associated with higher response rates and a longer median survival compared with chemotherapy alone (Slamon *et al*, 2001). Although effective, the combination of doxorubicin-cyclophosphamide and trastuzumab resulted in a higher percentage of severe cardiac dysfunctions compared with paclitaxel and monoclonal antibody. This observation and results of preclinical studies led to the development of combinations of trastuzumab and other antiblastic drugs, such as cisplatin (Pegram *et al*, 1998), vinorelbine (Burstein *et al*, 2001), taxotere (Esteva *et al*, 2002), liposomal doxorubicin (Theodoulou *et al*, 2002) or weekly paclitaxel (Seidman *et al*, 2001). Trastuzumab and weekly administration of paclitaxel in metastatic breast cancer with HER2 overexpression or normal HER2 expression have been evaluated, with promising results (Seidman *et al*, 2001).

In this phase II study, we evaluated the activity and toxicity of weekly trastuzumab and paclitaxel in HER2-overexpressing metastatic breast cancer patients, who had received prior therapy with anthracyclines and taxanes.

## PATIENTS AND METHODS

### Eligibility

Patients with metastatic, anthracycline- and taxane-pretreated breast cancer that overexpressed HER2 were considered for enrolment. Informed consent was obtained from all patients.

The eligibility criteria included a life expectancy ≥3 months, ECOG performance status ≤2, measurable lesions, adequate liver, renal and bone function, and normal left-ventricular ejection fraction (LVEF) measured by echocardiography.

Patients were excluded for active infection, pregnancy or lactation, significant cardiac disease (New York Heart Association Functional Class III or IV) or haemorrhagic diathesis, history of grade 3 or 4 peripheral neuropathy of any aetiology, osteoblastic bone metastases or pleural effusion or ascites as the only evidence of disease, a second type of primary cancer (except for carcinoma *in situ* of the cervix or nonmelanoma skin cancer), previous therapy with a monoclonal or polyclonal antibody or concomitant use of any investigational agent.

Tumour expression of HER2 was determined by immunohistochemical (IHC) analysis using a monoclonal antibody CB11 (Biogenex, San Ramon, CA, dilution 1 : 10, in 19 patients) or a polyclonal antibody DA485 (DAKO Corp., dilution 1 : 100, in six patients) on paraffin-embedded, formalin-fixed tumour specimens collected either at the time of initial biopsy or at recurrence. In order to be eligible for the study, all tumours had to have a weak to strong membrane staining in ≥60% of the neoplastic cells. Subsequently, we have reanalysed our data utilising the HercepTest scoring system from 0 to 3+ according to standard criteria, as follows: 0, if membrane staining was absent or present in less than 10% of tumour cells; 1+, if membrane positivity was weak and incomplete in more than 10% of tumour cells; 2+, if membrane positivity was moderate and complete in more than 10% of tumour cells; 3+, if more than 10% of tumour cells showed strong and complete membrane staining. Slides from 21 patients were reanalysed; in the other four patients, the slides for review were not available.

### Treatment

Before starting therapy, all patients had a complete staging that included history and physical examinations, blood count and chemistry profile, chest X-ray or CT, bone scan, liver ultrasound or CT. A bone X-ray or MRI survey was performed if abnormal uptake areas were observed in the bone scan. ECG and echocardiography were performed at baseline and then every 3 months.

Trastuzumab was administered intravenously (i.v.) in the outpatient setting at a loading dose of 4 mg kg^−1^ on day 0, followed by weekly administration of 2 mg kg^−1^. The first infusion was given over 90 min; if the infusion was well tolerated, subsequent infusions were shortened to 30 min. If patients experienced an infusion syndrome, treatment was stopped and a supportive treatment was started if necessary (acetaminophen, dexamethasone, diphenhydramin, H2 antagonist).

Paclitaxel was administered at a dose of 60–90 mg m^−2^ over 1-h i.v. infusion. The starting dose was determined on the basis of the prior treatment (type of antineoplatic drugs, number of previous chemotherapy regimens and prior toxicities), age and performance status of the patient. The first dose of paclitaxel was given on day 2 and then weekly on the same day as and after trastuzumab administration. Premedication consisted of dexamethasone 10 mg i.v., orphenadrine 40 mg i.m., ranitidine 100 mg i.v. and ondansetron 8 mg i.v., all given 30 min before the paclitaxel infusion. Paclitaxel dose was adjusted each week on the day of therapy, based on haematologic toxicity. Paclitaxel was administered at full dose if the absolute neutrophil count was >1500 cells *μ*l^−1^ and/or platelet count >100 000 cells *μ*l^−1^. The drug was reduced by 50% if the absolute neutrophil count was 1499–1000 cells *μ*l^−1^ and/or the platelet count 99 000–50 000 cells *μ*l^−1^; paclitaxel was omitted if the absolute neutrophil count was <1000 cells *μ*l^−1^ and/or the platelet count <50 000 cells *μ*l^−1^. No dose adjustments for trastuzumab therapy were considered.

After 6 months of therapy, the treatment was left to the discretion of physicians. In patients with responsive or stable disease, the protocol allowed the investigators to continue paclitaxel and trastuzumab until progression or prohibitive toxicity, or to stop paclitaxel while continuing trastuzumab. When disease progression was detected, weekly trastuzumab could be continued at the dose of 2 mg kg^−1^ or discontinued at the physician's discretion, because the utility of continuing trastuzumab in progressing patients is still uncertain. Additional antitumour therapy was also permitted upon disease progression.

### Tumour response and statistical methods

Response to therapy was assessed every 12 weeks of treatment, according to the WHO criteria (Miller *et al*, 1981). Toxicity evaluation was performed every week and graded according to the National Cancer Institute Common Toxicity Criteria (NCI-CTC).

The primary end points were tumour response rate and toxicity of treatment. The secondary end points were duration of response, time to progression and survival.

Duration of response was defined as the time from the start of therapy to disease progression. Time to progression (TTP) was defined as the time from the start of treatment to disease progression or death (whichever occurred first), and was censored at the last date of contact for patients whose disease did not progress. Survival was defined as the time from the start of therapy to death, and was censored at the date of last contact for patients who were alive.

Responses, TTP and survival were determined for all eligible enrolled patients. The time to event end points was estimated by Kaplan–Meier survival methodology (Kaplan and Meier, 1958).

## RESULTS

From November 1999 to July 2001, 25 patients with HER2-overexpressing metastatic breast cancer and pretreated with anthracycline-containing regimens entered this phase II trial. Patient baseline characteristics are listed in Table 1

**Table 1:** Patient characterists (*n*=25)

		**Patients**	
**Characteristic**		**No.**	**%**
Median age (range)	43 years (29–64)		
			
Median ECOG PS (range)	0 (0–1)		

*Receptor status*			
ER and PgR negative		13	52
ER and/or PgR positive		11	44
Unknown		1	4

*No. of metastatic sites*			
1		3	12
2		14	56
≥3		8	32

*Metastatic site*			
Soft tissue		2	8
Bone		1	4
Visceral		22	88

*Prior therapy*			
Adjuvant chemotherapy		23	92
Metastatic chemotherapy		19	76
One prior regimen		7	28
≥2 prior regimens		12	48

Prior anthracyclines		25	100

Prior taxanes		20	80

Bone marrow or stem cells transplantation		2	8

*Disease-free interval*			
0–12 months		6	24
13–24 months		7	28
>24 months		12	48

.

A total of 20 patients had already received taxanes (docetaxel or paclitaxel in combination with other drugs): nine patients received paclitaxel (six patients in metastatic, one patient in adjuvant and two patients in neoadjuvant setting) and 11 patients docetaxel (all in metastatic setting). In metastatic setting, the median interval from prior taxane to enrollment in the study was 6.5 months (range 5–52 months) for paclitaxel and 5 months (range 2–21 months) for docetaxel; objective responses were seen in 33% of patients who received paclitaxel and in 27% of patients who received docetaxel, respectively. The time to recurrence from prior paclitaxel therapy was 10 and 30 months for the two patients treated in neoadjuvant setting and 10 months for the only patient who received paclitaxel in adjuvant setting.

In all, 15 patients (60%) were postmenopausal and 22 (88%) had visceral involvement. A total of 19 patients (72%) had received chemotherapy for advanced disease.

A total of 528 weekly trastuzumab and paclitaxel infusions were delivered with a median of 24 weekly treatments per patient (range 6–45). The paclitaxel median delivered dose was 70 mg m^−2^ week^−1^ (range 60–90 mg m^−2^).

All patients were assessable for response. The overall response rate was 56% (95% CI, 36.5–75.5%) with four complete responses (16%) and 10 partial responses (40%). Stable disease was observed in 16% of patients. Only seven patients had progression disease (28%). A response was obtained in 11 of 20 patients pretreated with taxanes (55%). The median response duration was 10.4 months (range 4.1–24.2+).

In the four patients with complete response, the weekly monotherapy with trastuzumab was continued after 25, 29, 37 and 45 doses of trastuzumab and paclitaxel. In six out of 10 patients who had obtained a partial response after 6 months of trastuzumab and paclitaxel, trastuzumab was continued as monotherapy or in association with other antineoplastic drugs when disease progression was detected. In one out of four patients with stable disease, trastuzumab was continued for a total of 69 weekly doses at the date of this analysis.

When the herceptest scoring was applied, 67% of tumours were HER2 3+, 29% HER2 2+ and 5% HER2 1+. This retrospective analysis was performed in 11 out of 14 responsive patients: the tumours were 3+ in 10 responsive patients (91%) and 2+ in one patient (9%).

At the median follow-up of 19.6 months (range 9.2–38.1), the median time to progression was 8.6 months (range 2.5–24.2+); only nine patients died and the median overall survival has not been reached.

In six out of 14 responsive patients (42.8%), the relapses occurred in the central nervous system (CNS) and in five patients (83.3%) the brain metastases represented the only site of progressive disease, while complete or partial response was maintained in the other disease sites. These patients continued trastuzumab therapy during and after whole-brain irradiation.

### Toxicity

Treatment was generally well tolerated (Tables 2

**Table 2:** Toxicity-NCI criteria (25 assessable patients)

	**Events, *n* (%)**			
	**Grade 1**	**Grade 2**	**Grade 3**	**Grade 4**
*Haematologic*				
Anaemia	2 (8%)	2 (8%)	—	—
Leukopenia	1 (4%)	1 (4 %)	—	—
Thrombocytopenia	—	—	—	—
				
*Gastrointestinal*				
Nausea	3 (12 %)	—	—	—
Vomiting	2 (8%)	—	—	—
Stomatitis	1 (4%)	2 (8%)	—	—
Diarrhoea	—	—	—	—
Alopecia (assessable in 14 pts)	6 (43%)	5 (36%)	—	—
Neurosensory	6 (24%)	3 (12%)	—	—
Cardiovascular	—	—	2 (8%)	—
Myalgia	3 (12%)	—	—	—
Asthenia	3 (12%)	—	—	—
Hypersensitivity reaction	2 (8%)	2 (8%)	1 (4%)	—
Onycholysis	—	5 (20%)		—

and 3

**Table 3:** Response to therapy

	**Patients**	
**Response**	**No.**	**%**
Overall response (CR+PR)	14	56*
Complete response	4	16
Partial response	10	40
Stable disease	4	16
Progressive disease	7	28

* 95% CI, 36.5–75.5%.

). Hypersensitivity reactions including fever and chills were recorded in five patients during the first infusion of trastuzumab, and in one patient it was associated with symptomatic bronchospasm. Leukopenia grade 2 was observed in one patient (4%) and anaemia grade 2 in two patients (8%). Peripheral neuropathy grade 1–2 affected nine patients (36%); onycholysis grade 2 was observed in five patients (20%). Alopecia grade 2 was reported in 43% of 14 assessable patients.

Grade 3 cardiotoxicity was manifested in two patients (8%) after 9 and 17 weeks of treatment, respectively. These patients developed symptomatic congestive heart failure with decline in left ventricular ejection fraction in one patient (EF=35%). Cardiac function improved with standard medical therapy, but the patients came off study. Both had already received anthracyclines and taxanes for metastatic disease.

## DISCUSSION

The results of this phase II study suggest that treatment with weekly trastuzumab and paclitaxel is very active in patients with HER2-overexpressing metastatic breast cancer, who have previously received anthracycline-based regimens.

We obtained an overall response rate of 56%, comparable with that reported in HER2 normal and HER2-overexpressing pretreated patients (56.8%) utilising weekly trastuzumab and paclitaxel (Seidman *et al*, 2001). Similar results (62% of objective responses) were also obtained with the same combination administered as first-line in 35 patients with HER2-overexpressing metastatic breast cancer (Fountzilas *et al*, 2001). However, in our study, the majority of patients (76%) were pretreated with chemotherapy for advanced disease and 48% of patients had received ⩾2 prior regimens.

Interestingly, 11 out of 20 patients (55%) pretreated with taxanes responded to weekly trastuzumab and paclitaxel. Overexpression of HER2 blocks paclitaxel-induced apoptosis by upregulation of a cycline-dependent kinase inhibitor (p21), which inhibits p34^cdc2^ kinase (Yu *et al*, 1998). Probably, trastuzumab, by regulating HER2, inactivates the p21 and restores sensitivity to paclitaxel-induced apoptosis.

In our experience, the median response duration was 10.4 months (range 4.1–24.2+); at the median follow-up of 19.6 months (range 9.2–38.1), the median time to progression was 8.6 months (range 2.5–24.2+) and median overall survival had not been reached. This could probably be due to the fact that, after 6 months of therapy, treatment with trastuzumab was continued in 11 patients, and this could have positively affected the survival.

In the present study, no specific method of IHC was recommended to estimate HER2 overexpression. Two antibodies (monoclonal and policlonal) were used. Due to the small number of patients, it is impossible to compare the responsiveness according to each method. For the trial, we have considered tumours with a weak to strong membrane staining in ≥60% of the neoplastic cells, because accumulating evidence suggests that the degree of HER2 overexpression is important for prediction of response to trastuzumab. When the herceptest scoring system was applied, the tumours resulted IHC 3+ in 91% of responsive patients whose tissue samples were available, and IHC 2+ in 9%. These results reflect the problem of the more appropriate methods to evaluate the HER2 status.

In our trial, the haematological, gastrointestinal and neurological toxicities were mild. Since we had decided to include in our trial anthracycline- and taxane-pretreated patients, we planned a dose reduction schema for haematologic toxicity for paclitaxel. Actually, we observed only grade 1 and 2 haematologic toxicity in a few patients, and we think that our dose reduction schema is excessive and unnecessary. The neurotoxicity was also very mild (only grade 1 and 2). The median dose of paclitaxel in our experience was 70 mg m^−2^ week^−1^, lower than that used by Seidamn *et al* (91 mg m^−2^ week^−1^) (Seidman *et al*, 2001). The difference could explain the absence of grade 3–4 neurotoxicity in our patients, pretreated also by taxanes. We observed grade 3 cardiotoxicity in two out of 25 patients (8%) pretreated with anthracyclines, comparable with the results reported by other authors (Seidman *et al*, 2002). Both our patients had received a cumulative dose of epidoxorubicin >400 mg m^−2^, which is the only risk factor identified for cardiotoxicity, besides the concomitant use of anthracycline and advanced age (Slamon *et al*, 2001); these two patients were also pretreated with taxanes for metastatic disease. To date, we do not know the true incidence of trastuzumab cardiac toxicity when it is given alone or with other antineoplastic drugs; the ongoing clinical trials, with careful cardiac monitoring, are likely to give us more accurate information Speyer, 2002. The mechanism of trastuzumab cardiotoxicity is unknown (Ewer *et al*, 1999; Keefe *et al*, 2002; Seidman *et al*, 2002) and the pathogenesis and histologic changes responsible for trastuzumab-associated cardiotoxicity are currently under investigation. Understanding the mechanisms of the trastuzumab-associated cardiotoxicity is important to provide strategies for the prevention or identification of patients at risk for cardiac complications. Given the poor prognosis of patients with HER2-overexpressing metastatic breast cancer, the cardiotoxicity of trastuzumab must be weighed against its potential clinical benefit. As these results confirm that weekly administration of trastuzumab and paclitaxel is active in anthracycline-pretreated metastatic breast cancer HER2-overexpressing patients (RO=56%), and in the subset of patients also pretreated with taxanes (RO=55%), this risk of cardiac dysfunction (8%) can be considered acceptable. Anyway, we recommend that cardiac function should be established at baseline and monitored regularly (every 3–4 months) during treatment by physical examination and measurement of the left-ventricular ejection fraction (by MUGA scan or echocardiography). This is very important because some patients who develop cardiotoxicity while receiving treatment with trastuzumab recover systolic function with medical standard therapy, and are able to continue trastuzumab therapy.

We observed a high incidence of relapse (42.8%) in CNS: in five patients (83.3%), the brain metastases represented the only site of progressive disease, while complete or partial response was maintained in the other disease sites. The incidence of this complication has also been reported by other authors (Lower *et al*, 2001). The high incidence of developing CNS metastases may be increased in these patients because of their prolonged survival. Moreover, during intravenous trastuzumab treatment, only a minimal amount of monoclonal antibody penetrates the cerebrospinal fluid (CSF) (Pestalozzi *et al*, 2000) as well as taxanes.

In conclusion, the present trial demonstrates that weekly trastuzumab and paclitaxel is a safe and effective treatment in anthracycline- and taxane-pretreated metastatic breast cancer patients. The addition of trastuzumab to chemotherapy positively affects the natural history of the HER2-overexpressing breast cancer, though many questions about the best dose and schedule (weekly or 3-weekly) of trastuzumab (Gelmon *et al*, 2001; Carbonell *et al*, 2002), the best combination and the optimal duration of therapy with trastuzumab need to be addressed. There is no information about a comparison of trastuzumab and chemotherapy *vs* trastuzumab as single-agent therapy, followed by chemotherapy at the time of progression. To date, the combined approach is the only one that has shown a survival gain compared with chemotherapy alone (Slamon *et al*, 2001), but the sequential approach would be more cost-effective (early identification of patients who do not benefit from trastuzumab) and could delay, in the trastuzumab-responsive patients, the side effects related to chemotherapy (Burris *et al*, 2000).
